# Correction to “A Microcycle of High‐Intensity Short‐Interval Sessions Induces Improvements in Indicators of Endurance Performance Compared to Regular Training”

**DOI:** 10.1002/ejsc.70241

**Published:** 2026-08-18

Solli, G. S., I., Odden, V., Sælen, J., Hansen, K. S., Mølmen. and B. R., Rønnestad. 2025, “A microcycle of high‐intensity short‐interval sessions induces improvements in indicators of endurance performance compared to regular training.” *European Journal of Sport Science* 25: e12223. https://doi.org/10.1002/ejsc.12223.

This paper was initially tagged as a “Review”. This has now been corrected to “Original Paper”.

We apologize for this error.

